# History-dependent changes to distribution of dominance phases in multistable perception

**DOI:** 10.1167/jov.23.3.16

**Published:** 2023-03-28

**Authors:** Alexander Pastukhov, Malin Styrnal, Claus-Christian Carbon

**Affiliations:** 1 Department of General Psychology and Methodology, University of Bamberg, Bamberg, Bavaria, Germany; 2 Research Group EPÆG (Ergonomics, Psychological Æsthetics, Gestalt), Bamberg, Bavaria, Germany

**Keywords:** multistable perception, bistable perception, autocorrelation, gamma distribution, serial dependence, history dependence, binocular rivalry, Necker cube, kinetic-depth effect, Bayesian statistics

## Abstract

Multistability – spontaneous switches of perception when viewing a stimulus compatible with several percepts – is often characterized by the distribution of durations of dominance phases. For continuous viewing conditions, these distributions are similar for various multistable displays and share two characteristic features: a Gamma-like distribution shape and dependence of dominance durations on the perceptual history. Both properties depend on a balance between self-adaptation (also conceptualized as a weakening stability prior) and noise. Prior experimental work and simulations that systematically manipulated displays showed that faster self-adaptation leads to a more “normal-like” distribution and, typically, to more regular dominance durations. We used a leaky integrator approach to estimate accumulated differences in self-adaptation between competing representations and used it as a predictor when fitting two parameters of a Gamma distribution independently. We confirmed earlier work showing that larger differences in self-adaptation led to a more “normal-like” distribution suggesting similar mechanisms that rely on the balance between self-adaptation and noise. However, these larger differences led to less regular dominance phases suggesting that longer times required for recovery from adaptation give noise more chances to induce a spontaneous switch. Our results also remind us that individual dominance phases are not “independent and identically distributed.”

## Introduction

**Figure 1.**
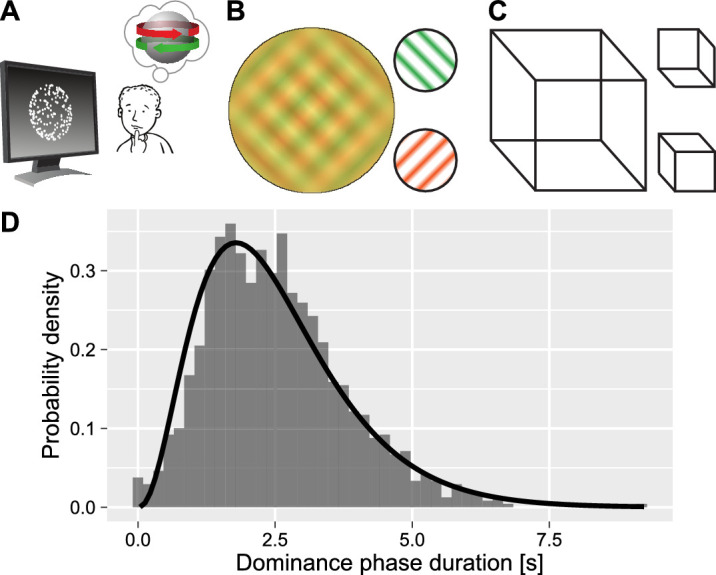
Bistable displays and typical distribution of dominance phase durations. (**A**) The kinetic-depth effect: a 2D onscreen motion produces an alternating perception of a rotating object. (**B**) Monocular rivalry generates alternating dominance of two orthogonal gratings (use red-green glasses to experience binocular rivalry). (**C**) A Necker cube can be perceived in two ways with different orientations. (**D**) A typical distribution of dominance phases for a participant who viewed kinetic-depth effect displays. Data is overlaid by a fitted Gamma distribution.

Perception is constructed from intrinsically noisy and ambiguous sensory information, so our brain uses prior knowledge about the world to “fill in the gaps” and resolve ambiguity (Carbon, 2014). In some cases, sensory information is compatible with different, similarly probable perceptual interpretations leading to so-called multistability, meaning our perception spontaneously alternates despite a constant physical stimulus (Tong, Meng, & Blake, 2006). Examples of multistable stimuli can be seen in Figures 1A–C, and there exist many stimuli beyond the visual domain, including auditory (Denham & Winkler, 2006), tactile (Liaci, Bach, Van Elst, Heinrich, & Kornmeier, 2016), and even olfactory multistable stimuli (W. Zhou & Chen, 2009). Multistable perception is also not uniquely human and has been demonstrated in primates (Leopold & Logothetis, 1996), pigeons (Vetter, Haynes, & Pfaff, 2000), and mice (Zhang, Wen, Zhang, She, Wu et al., 2012). Due to its universality, multistable perception is a particularly interesting subject of research as it advances our knowledge about the architecture of our perceptual system and perceptual decision making (Cao, Pastukhov, Aleshim, Mattia, & Braun, 2021).

When multistable displays are presented continuously, their perception is often characterized by a distribution of dominance duration times or, inversely, by the alternation rate. These have two properties that are of interest here: the overall shape of the distribution and their dependence on the perceptual history. With respect to the former, even though duration times vary greatly among participants (Brascamp, Becker, & Hambrick, 2018; Pastukhov, Kastrup, Abs, & Carbon, 2019), the shape of individual distributions is remarkably similar (Cao, Pastukhov, Maurizio, & Braun, 2016), see Figure 1D. This consistency is viewed as a “hallmark” of multistable perception (Leopold & Logothetis, 1999) and is often used as a first check when characterizing a new multistable stimulus (van Ee, 2005). There is a debate about which theoretical distribution fits the empirical dominance phase durations/alternation rate the best, with many suggestions that include Gamma, exponential, Weibull, normal, Capocelli-Ricciardi, beta rate, exponentially modified Gaussian, and log-normal distributions (Brascamp, van Ee, Pestman, & van den Berg, 2005; Cogan, 1973; de Marco, Penengo, & Trabucco, 1977; Huguet, Rinzel, & Hupé, 2014; Lehky, 1995; Levelt, 1967; Zhou, Gao, White, Yao, & Merk, 2004). Among those, the Gamma distribution is considered to be the “canonical” distribution for fitting the data, which is described by two parameters, the shape and scale parameters (Borsellino, De Marco, Allazetta, Rinesi, & Bartolini, 1972; Levelt, 1967; Murata, Matsui, Miyauchi, Kakita, & Yanagida, 2003). Its initial appeal was that it allows conceptualizing perceptual alternations as a Poisson process so that they occur after α discrete independent stochastic events (Borsellino et al., 1972; Levelt, 1967). This conceptualization predicts that the shape parameter of the Gamma distribution corresponds to that number of discrete independent stochastic events and therefore must be an integer number (Murata et al., 2003), but the evidence for this is not conclusive.

Although the conceptualization of alternations as a Poisson process assumes independence of events, dominance phase durations exhibit a subtle but consistent dependence of their duration on the perceptual history. This serial dependence is particularly evident when an unambiguous stimulus precedes a bistable one. Here, an artificially prolonged dominance of a percept leads to longer dominance of the alternative percept during a consequent continuous presentation (Blake, Westendorf, & Fox, 1990; Wolfe, 1984). Although less pronounced, the same serial dependence is observed when fully ambiguous bistable displays are viewed continuously. A common approach to quantify this dependence is via an autocorrelation of dominance phase durations with different lags (Borsellino et al., 1972). Typically, this produces a small (0.1–0.2) but significant and consistently positive autocorrelation for lag 1 (van Ee, 2009). In other words, longer dominance phases for one percept tend to be followed by similarly long dominance phases for the complementary percept, and, conversely, short perceptual dominance phases are typically followed by similarly short ones. Alternatively, the effect of the perceptual history can be quantified via a leaky integrator that accumulates perceptual history for each state (Pastukhov & Braun, 2011). The latter approach requires fitting a decay time parameter. However, it is more robust against return transitions (events when the same perceptual state dominates again after a brief mixed phase period) and erroneous key presses and, therefore, was adopted for the current study.

There are numerous computational models of multistable perception that reproduce both the characteristic distribution of dominance phases and their history-dependence (Cao et al., 2016; Cao et al., 2021; Kornmeier, Friedel, Wittmann, & Atmanspacher, 2017; Laing & Chow, 2002; Moreno-Bote, Rinzel, & Rubin, 2007; Noest, van Ee, Nijs, & van Wezel, 2007; Pastukhov, García-Rodríquez, Haenicke, Guillamon, Deco et al., 2013; Shpiro, Moreno-Bote, Rubin, & Rinzel, 2009; Weilnhammer, Stuke, Hesselmann, Sterzer, & Schmack, 2017). One popular class of dynamic models assumes that multistability is borne out as an interplay among cross-inhibition (to ensure perceptual exclusivity), self-adaptation (gradual habituation of a dominant state through neural fatigue), and noise (Laing & Chow, 2002; Moreno-Bote et al., 2007; Noest et al., 2007; Pastukhov et al., 2013). Alternatively, perceptual disambiguation and ensuing instability of multistable perception can be conceptualized as inference via hierarchical predictive coding (Staadt, Philipp, Cremers, Kornmeier, & Jancke, 2020; Weilnhammer et al., 2017). Notably, both classes of models predict serial dependence due to leaky accumulation of a dominant state signal. In the former case, it is assumed to reflect noisy self-adaptation (van Ee, 2009), whereas in the case of the predictive coding framework, it corresponds to a weakening stability prior and an escalation of a prediction error (Weilnhammer et al., 2017). In the text below, we will use the terms “accumulated perceptual history” and “self-adaptation” interchangeably to refer to this phenomenon of history accumulation without implying a specific neural mechanism or conceptualization that it might reflect.

Both classes of models suggest that the shape of dominance phase durations and their regularity reflect the balance between the speed of accumulation and the level of noise. Specifically, the shape of their distribution can range from an exponential, when alternations are triggered by noise, to a more normal-like shape (i.e. a more symmetric distribution with less probability mass in the rightward tail), when self-adaptation is the main source of stabilization and destabilization (Pastukhov et al., 2013). The latter oscillatory regime also leads to more regular (i.e. predictable) dominance phase durations, whereas the noise-driven regime reduces autocorrelation. These predictions are mirrored by experimental evidence that shows, for example, that higher contrast in binocular rivalry (presumed to increase absolute stimulus strength and, therefore, to increase the effective speed of self-adaptation) leads to both a more normal-like shaped distribution (i.e. Gamma distributions with *higher* shape parameter values) and more regular dominance phase durations (van Ee, 2009). Conversely, an intermittent presentation that allows for recovery from self-adaptation leads to a more erratic noise-driven perception with exponential-like distributions (i.e. Gamma distributions with shape parameter values close to 1; Brascamp, Pearson, Blake, & van den Berg, 2009).

**Figure 2.**
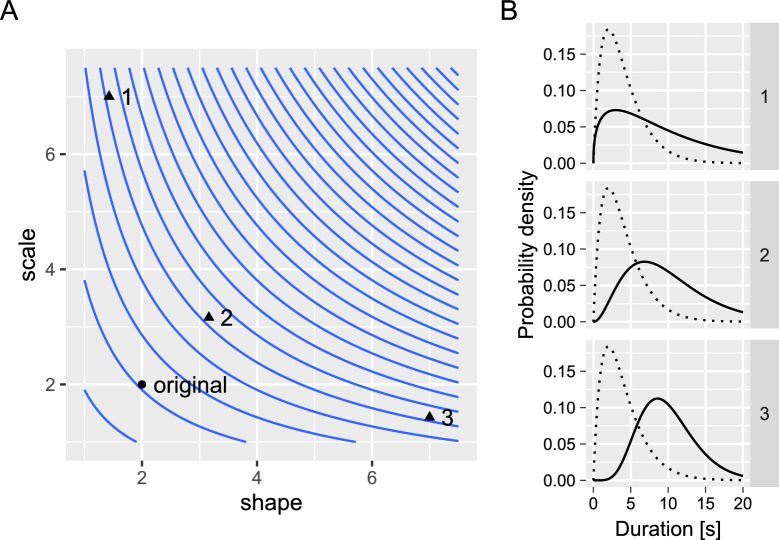
The same change of the mean of the Gamma distribution can come about as various combinations of changes to its shape and scale parameters. (**A**) Isolines indicate parameters’ combinations that produce the same mean. (**B**) Three example distributions that have the same higher mean as compared to the original (*dashed line*). Lower values of the shape parameter lead to more exponential-like distributions, whereas higher values lead to more normal-like distributions.

The prior work listed above hints at a relationship between the overall distribution shape and the regularity of dominance phases. However, in all cases, the balance between accumulation (self-adaptation) and the noise was fixed for an entire experimental or simulation run. Here, we sought to confirm and extend these results to minute dynamic changes in the balance due to recent perceptual experience, allowing us to compare different operating regimes naturally occurring within the time series. Our approach was two-fold. First, we fitted the Gamma distribution using linear models for each parameter. These models included accumulated perceptual history as a predictor, allowing them to change and adjust the overall distribution shape independently. This extends prior work that used accumulated perceptual history to model changes in the mean of the distribution (Borsellino et al., 1972; Pastukhov & Braun, 2011; van Ee, 2009), as the latter approach is limited as it does not inform on how a particular change of the mean originates, see Figure 2. In our case, linear models for both parameters of the Gamma distribution on the perceptual history allowed us to characterize its dynamic changes and compare them to changes due to fixed regimes cited above using both behavioral data and model simulations. Second, we computed the regularity (predictability) of dominance phase durations for different levels of accumulated perceptual history. This allowed us to compare them with regularity expected from stationary regimes both within the study, using contrast-manipulated stimuli as a benchmark, and with prior work.

## Materials and methods

### Behavioral data

We used a previously published data set (Pastukhov et al., 2013) and previously unpublished data (see the Table 1 and the description below). The first data set, reported in Pastukhov et al. (2013), contains results for binocular rivalry (BR), kinetic-depth effect (KDE), and Necker cube (NC) displays measured in an adult population. For the measurement method details, please refer to Pastukhov et al. (2013).

**Table 1.** Summary of data sets used in the present study. BR = binocular rivalry; KDE = kinetic depth effect display; NC = Necker cube.

	Source	Data set name	Display	Contrast (BR only)	Number of participants	Number of dominance phases
#1	(Pastukhov et al., 2013)	BR	BR	50%	8	6698
		KDE	KDE	–	11	43,456
		NC	NC	–	5	3940
#2	New data	Contrast	BR	6.25%	6	930
				12.5%		980
				25%		1000
				50%		1258
				100%		1296
	Total number of dominance phases					59,558

For the new data set, we used a binocular rivalry display and a procedure similar to that used in Pastukhov et al. (2013) but with five contrast levels 6.25%, 12.5%, 25%, 50%, and 100% (Michelson contrast, Gamma corrected, presented on Iiyama Vision Master Pro 454 display, luminance profile was measured using Konica Minolta LS-110 luminance meter). Binocular rivalry stimulus consisted of two orthogonally oriented sine wave gratings (radius 0.9 degrees, spatial frequency 2 cycles/degrees, one was tilted by 45 degrees clockwise and another by 45 degrees counter-clockwise). Participants viewed the display through a custom-made mirror stereoscope (75 cm, about 2.46 feet, eye-screen distance) and reported on the eye dominance using a keyboard, continuously pressing *left* or *right* when, correspondingly, the counterclockwise- or clockwise-oriented grating was dominant. The lack of key presses indicated mixed/transition phases. Each presentation lasted for 2 minutes. An experimental session consisted of 10 blocks so that each contrast condition was repeated twice. The order was randomized so that the five contrast conditions were shown in the random order and then again in the reverse order.

The data were collected at Otto von Guericke University Magdeburg. Six participants took part in the experiment. All participants signed the informed consent prior to the experimental session and received monetary compensation. They had normal or corrected-to-normal vision. All procedures were in accordance with the national ethical standards on human experimentation and with the Declaration of Helsinki of 1975, as revised in 2008, and were approved by the medical ethics board of the Otto-von-Guericke Universität, Magdeburg: “Ethikkommission der Otto-von-Guericke-Universität an der Medizinischen Fakultät.”

### Simulated data

**Figure 3.**
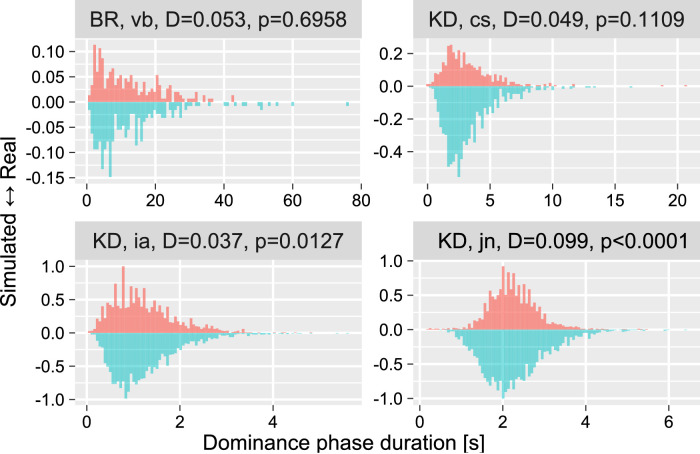
Example distributions of dominance phase durations for the experimental (upward) and matching simulated (downward) data for four participants. We picked four participants based on the Kolmogorov-Smirnov test *p* value from best-matched (BR, vb) to worst-matched (KD, jn) with two intermediate cases (at the second and third quartiles). The title above each plot shows the display, participant code, Kolmogorov-Smirnov test statistics, and the respective *p* value.

We generated simulated data using a custom implementation of a spiking neural model of bistability (Laing & Chow, 2002) based on the code provided by Stepan Aleshin and Jochen Braun from the Cognitive Biology group at Otto von Guericke University Magdeburg. The model was fitted to match the time series for each participant and display from the first data set (Pastukhov et al., 2013). We used a genetic algorithm (Scrucca, 2013) with the Kolmogorov-Smirnov test as a fitness function to match distributions of dominance phases for the experimental and the simulated data as closely as possible. Note that the fitness function characterized only the overall shape of the distribution and did not include any explicit measure for the history dependence. For the genetic algorithm, the population size was 50, and the number of iterations was 100. After the final iteration, we used parameters of the best-fitting model to generate a time series with 1100 clear percepts, but the first 100 percepts were discarded to account for the initial “burn-in” period of the model, see Laing and Chow (2002). See Figure 3 for examples of simulated data.

### Cumulative perceptual history

An accumulation of perceptual dominance history for each state was estimated via a leaky integrator with an exponential kernel (Pastukhov & Braun, 2011). Specifically, it was computed as a homogenous first-order process:

(1) $$\begin{array}{c} \frac{dh_{i}}{dt}=\frac{1}{\tau }\left(-h_{i}+S_{i}\left(t\right)\right)\;\; \end{array}$$

where τ is the time constant, h_i_ and S_i_(t) are, respectively, cumulative history and strength of a perceptual state *i* at time *t*. The latter was defined as:

(2) $$\begin{array}{c} \;\;\;\;\;\;\;\;\;\;\;\;S_{i}\left(t\right)=\left\{\begin{array}{cc} 1 & if\;state\;i\;is\;dominant\\ 0 & if\;state\;i\;is\;suppressed\\ S_{mixed} & for\;a\;mixed\;or\;transition\;phase \end{array}\right.\; \end{array}$$

so that 0 ≤ S_mixed_ ≤ 1. In the study, we fixed S_mixed_ = 0.5 based on prior work (Pastukhov & Braun, 2011).

As we assumed a constant strength throughout each period, the solution is:

(3) $$\begin{array}{c} h_{i}\left(t+\Delta t\right)=S_{i}+\left(h_{i}\left(t\right)-S_{i}\right)\cdot e^{-\frac{\Delta t}{\tau }}\; \end{array}$$

The initial state of cumulative history for both states was assumed to be zero (h_i_(0) = 0), whereas the time constant τ was fitted (see below).

For bistable stimuli used in the current study, there are two perceptual states and two corresponding cumulative history variables. For the models below, we combined these two variables as:

(4) $$\begin{array}{c} \Delta h\left(t,\tau \right)=h_{suppressed}\left(t,\tau \right)-\;h_{dominant}\left(t,\tau \right)\; \end{array}$$

where *h*_dominant_ and *h*_suppressed_ are cumulative history values for currently dominant and suppressed percepts. For example, for binocular rivalry displays, if a right eye is currently dominant then *h*_dominant_ = *h*_right_ and *h*_suppressed_ = *h*_left_.

### Statistical models

The data from Pastukhov et al. (2013), the contrast-manipulation data, and simulated data were fitted using a *bistablehistory* package (Pastukhov, 2022). We used identical models for both parameters of the Gamma distribution. Therefore, model descriptions below are for just one parameter (shape, *κ*).

The following model is the default model of the *bistablehistory* package. In the model below, *i* is the row index within the data table. Note that although cumulative history was computed using the entire time series, *Duration_i_* included only clear dominant states.

$$\begin{array}{c} Duration_{i}∼Gamma\left(k_{i},\;\theta_{i}\right)\\ log\left(k_{i}\right)=\alpha_{P_{i\;}}+\beta_{H_{i}}\cdot H\left(T_{i},\;\tau_{i}\right)+\beta_{T}\cdot \log\left(T_{i}\right)\\ a_{P_{i}}∼Normal\left(\log\left(3\right),\;5\right)\\ \log\left(\tau_{i}\right)=\;\tau_{pop}+\tau_{P_{i}}\\ \tau_{pop}∼Normal\left(\log\left(1\right),\;0.15\right)\\ \tau_{P_{i}}∼Normal\left(0,\;\sigma_{\tau }\right)\\ \sigma_{\tau }∼Exponential\left(10\right)\\ \beta_{H_{i}}=\beta_{H}+\beta_{H_{P_{i}}}\\ \beta_{H}∼\;Normal\left(0,\;1\right)\\ \beta_{H_{P_{i}}}∼Normal\left(0,\;\sigma_{\beta_{H}}\right)\\ \sigma_{\beta_{H}}∼Exponential\left(1\right)\\ \beta_{T}∼Normal\left(0,\;1\right) \end{array}$$

We used independent intercept terms for each participant (α_*P*__*i*_) as prior work shows high variability between individual observers (Brascamp, Qian, Hambrick, & Becker, 2019; Cao et al., 2016), which were weakly regularized by a prior centered at 3 seconds (it is coded as log(3) due to the log link function). Each participant was assigned an individual value for both the time constant of cumulative history (τ_*i*_) and the slope term for the effect of cumulative history (β_*H*__*i*_) via a pooled multilevel approach. We used the log link function to ensure that τ_*i*_ is strictly positive and a strongly regularizing prior for both the population-level τ_*pop*_ and variability of individual participants. We used a neutral, weakly regularizing prior for the population-level effect of cumulative history β_*H*_ and strongly regularizing prior for variability of individual observers. The model included an effect of time via log (*T_i_*) to account for a slow overall trend within each run (Mamassian & Goutcher, 2005). We used a neutral weakly regularizing prior for β_*T*_. The code for the model is available online as part of the *bistablehistory* package. The same model was used for fitting KDE display data from Pastukhov et al. (2013) but with a stronger and more conservative prior $\tau_{pop}\;∼\;Normal\left(\log(0.5),\;0.3\right)$ as the default prior led to frequent divergent transitions.

The same model was used for fitting data for the BR stimulus with modulated contrast. It included an additional term for the log contrast in the linear model:

$$\begin{array}{ccc} log\left(k_{i}\right) & \;= & \alpha_{P_{i\;}}+\beta_{H_{i}}\cdot H\left(T_{i},\;\tau_{i}\right)+\beta_{T}\cdot \log\left(T_{i}\right)+\\ & & \beta_{C}\cdot log\left(C_{i}\right)\\ \beta_{C} & \;∼ & Normal\left(0,\;1\right) \end{array}$$

where *C_i_* was the stimulus log contrast.

### Reported statistics

We characterized individual model terms using the samples from the posterior distribution. For each term, we computed the mean and an 89% credible interval, a range that contains 89% of the probability mass based on values from the sampled posterior distribution (CI – credible interval, also called compatibility interval). The choice of CI is arbitrary and, therefore, we chose to use an 89% CI because 89 is a prime number.

All models use the log link function, therefore, reported values correspond to a multiplicative change in the outcome variable. For example, β_H_ corresponds to a 100 × β_H_% change in parameter value (either shape or scale) when cumulative history changes from 0 to 1. Similarly, β_T_ corresponds to a 100 × β_T_% change in parameter value for a one unit increase in log time. β_C_ corresponds to a 100 × β_C_% change in parameter value for a one unit increase in contrast.

### Software

The analysis and modeling were performed in R 4.1.2 (R Core Team, 2022) using the *Tidyverse* collection of packages (Wickham, Averick, Bryan, Chang, McGowan et al., 2019). We fitted the data using the *bistablehistory* package (Pastukhov, 2022). The spiking neural model of bistability was implemented using *Rcpp* (Eddelbuettel & François, 2011). We used the *GA* package (Scrucca, 2013) for genetic algorithm optimization.

### Open practices statement

Behavioral data is available via the *bistablehistory* package (Pastukhov, 2022). Simulated data, the model code, scripts for fitting the data and for the analysis, as well as sampled models are available under Creative Commons Attribution 4.0 International Public License at https://osf.io/js3wv.

## Results

As described in the Introduction, the purpose of the study was to investigate (1) how the perceptual history influences the overall shape of the distribution of dominance phase durations and (2) how different levels of accumulated perceptual history alter the regularity (predictability) of individual dominance phases. We tackled these two questions separately by looking at corresponding changes in both behavioral and simulated data.

### Changes to the overall shape of the distribution

#### Three perceptual displays: Behavioral data

**Figure 4.**
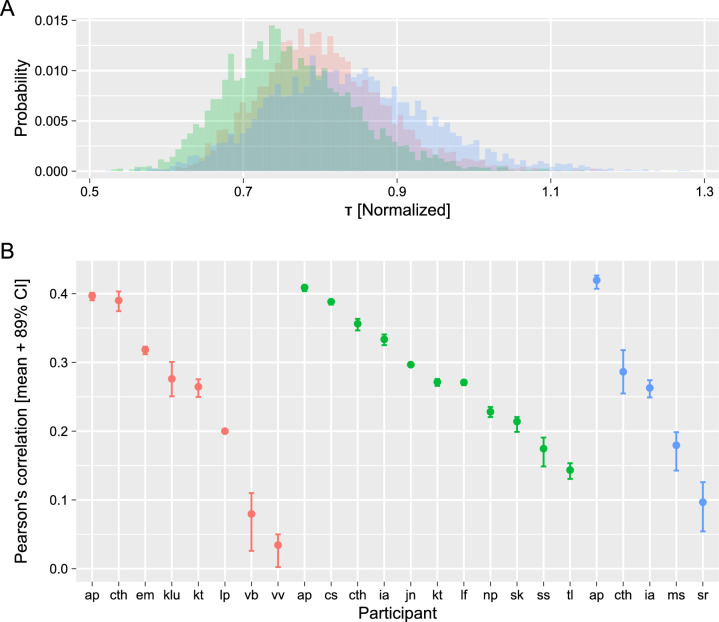
Estimates for accumulated perceptual history for three displays (Pastukhov & Braun, 2011). (**A**) Posterior distributions of accumulated history time constant. (**B**) Correlation between predicted and observed dominance phase durations for each participant (sorted by the correlation strength), mean, and 89% credible interval.

The first data set we used contained measurements of three different bistable displays (Pastukhov & Braun, 2011). In Pastukhov and Braun (2011), a cumulative history time constant τ was fitted by maximizing Pearson's correlation between an estimate of the accumulated perceptual history and the duration of the following dominance phase. This approach is equivalent to parameterization of the Gamma distribution via mean and variance with a linear model that computes a normalized and centered mean of the distribution. Here, we modified the loss function to accommodate for fitting both parameters of the Gamma distribution (shape and scale) to minimize the residual difference between the unscaled mean of the distribution and the following dominance phase duration (i.e. to maximize the strength of the correlation between predicted and observed durations). These two parametrizations produced comparable estimates for both an optimal cumulative time constant τ (Figure 4A) and the correlation between predicted and observed dominance durations (Figure 4B). In both cases, the correlation strength varied greatly between participants with a good agreement on the relative correlation strength between the studies (see Figure 3 in Pastukhov & Braun, 2011). Note that a quantitative comparison of correlation strength to Pastukhov and Braun (2011) is complicated but the fact that although the current approach used more parameters, it also used regularization via adaptive priors that both reduced effective degrees of freedom of the model and made estimates more conservative.

**Figure 5.**
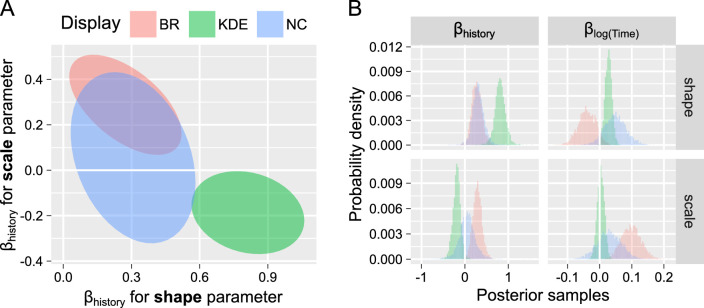
Posterior distributions for main effects of accumulated perceptual history and time for three displays (Pastukhov & Braun, 2011). (**A**) A joint posterior distribution for the effect of cumulative history (β_H_) for shape and scale parameters. (**B**) Marginal posterior distributions for effects of cumulative history (β_H_) and (log scaled) time (β_T_). See *Reported Statistics* Methods section for details on units.

With respect to the effect of the accumulated history on the distribution, we observed a clear and positive effect of cumulative history on the shape parameter (Figure 5). For all three displays, higher levels of accumulated perceptual history (i.e. a larger difference in levels of self-adaptation) increased the shape parameter leading to a more normal-like distribution. If larger differences in estimated accumulated perceptual history imply a shift of balance in favor of perceptual adaptation, these results are compatible with those listed in the Introduction. Namely, both simulation (Pastukhov et al., 2013) and behavioral (van Ee, 2009) studies show a similar change when balanced was shifted in favor of adaptation for the entire run. In contrast, we found neither a consistent effect of cumulative history on the scale parameter nor a consistent effect of (log) onset time. The latter lack of consistency is likely due to stimulus-specific speeding up and slowing down throughout an experimental run (Brown, 1955; Lehky, 1995; Suzuki & Grabowecky, 2007).

#### Contrast data set

**Figure 6.**
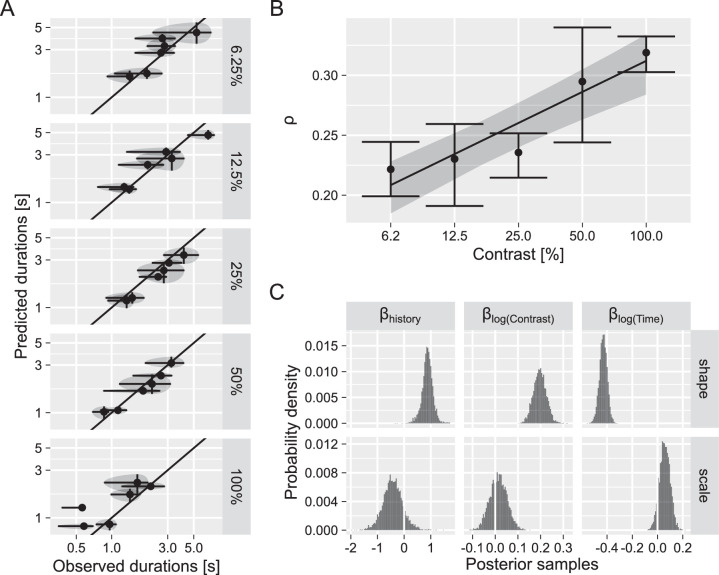
Effect of contrast in binocular rivalry stimulus on dominance phase durations. (**A**) Average observed and predicted durations of dominance phases, log scale for both axes. *Circles* depict the mean values, whereas *bars* and *shaded areas* depict the first and third quantiles for each participant. (**B**) Average group correlation between observed and predicted dominance durations as a function of contrast. Error bars depict 89% credible intervals. Line and stripe show mean and 89% credible interval for the linear regression with correlation strength as an outcome variable and contrast as a predictor variable. (**C**) Marginal posterior distributions for effects of cumulative history (β_H_), log contrast (β_C_), and log time (β_T_) on shape and scale parameters of the distribution.

Next, we analyzed data for a binocular rivalry display with different contrast levels ranging from 6.5% to 100%. Consistent with prior work (Brascamp, van Ee, Noest, Jacobs, & van den Berg, 2006; van Ee, 2009), we observed faster switching (shorter dominance durations; Figure 6A) as well as a stronger correlation between predicted and observed dominance durations (i.e. stronger dependence on prior perceptual history; Figure 6B) for higher contrast levels (more regular dominance durations). To quantify the latter, we fitted a linear regression model with contrast as a predictor and correlation strength as an outcome (ρ = α + β_*contrast*_ · log(*Contrast*) + ε) for each posterior sample, obtaining a posterior distribution for the slope term: β_*contrast*_ = 0.04 [0.03..0.05].

Concerning the effect of individual predictors on the parameters of the Gamma distribution (Figure 6C), we again found that higher values for accumulated perceptual history (larger differences in self-adaptation level) result in a significant increase in the shape parameter. Contrast also had a strong and positive effect on the shape parameter. However, as for the data set above, the effects of other parameters were mixed, with both accumulated history and contrast having a weak negative effect on the scale parameter. In short, results for the two data sets were qualitatively and quantitatively similar showing a shift toward a more “normal-like” distribution shape for higher levels of accumulated perceptual history (larger differences in self-adaptation level and more regular dominance phases).

#### Three perceptual displays: Simulated data

Finally, we performed the analysis on data simulated via a spiking neural model of bistability (Laing & Chow, 2002). We chose that particular model because it is frequently used as a starting (Noest et al., 2007) and reference point (Cao et al., 2021) for more complex models. Thus, its value was in investigating how well even such a basic model can reproduce changes in the distribution of dominance durations due to perceptual experience. To make this test more stringent, we deliberately excluded from optimization of any parameters related to history dependence. Instead, we used a genetic algorithm (Scrucca, 2013) to match the *overall* distribution of dominance phase durations as closely as possible using the Kolmogorov-Smirnov test as a fitness function (see examples in Figure 3). Thus, any effect of perceptual history on parameters of the Gamma distribution would not reflect model tuning. Nonetheless, we found that the simulated time series exhibited the history-dependence, although estimates of the cumulative history time constant were lower and less certain than those for the real observers: 0.93 (0.71..1.18; mean and 89% credible interval) for simulated data versus 0.8 (0.68..0.94) for behavioral data for BR, 0.81 (0.63..1.02) versus 0.76 (0.64..0.91) for KD, and 0.91 (0.71..1.14) versus 0.84 (0.68..1.02) for NC.

**Figure 7.**
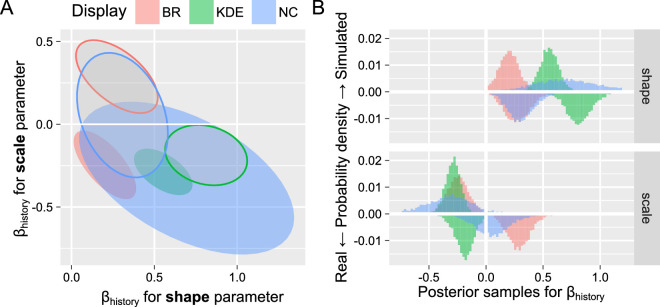
The posterior distribution for cumulative history term for simulated versus real data. (**A**) Joint posterior distribution for the effect of cumulative history (β_H_) for shape and scale parameters. Filled – simulated data, open – real data. (**B**) Marginal posterior distributions for effect of cumulative history (β_H_) for each parameter. Simulated data – positive direction, real data – negative direction.

We used the same fitting approach as for the behavioral data but excluded the (log) onset time predictor as the model does not generate any long-term trend (Laing & Chow, 2002). The results are summarized in Figure 7. Here, we observed a very consistent strong positive effect for the shape parameter (matching the behavioral data) but an equally strong and consistent *negative* effect for the scale parameter. The latter difference is likely to stem from the lack of a larger timescale trend typical for the behavioral data, as any increase in shape parameter must be offset by an opposite decrease in scale to keep the average dominance duration constant (see Figure 2). Therefore, it is likely that a similar effect on shape parameters in behavioral data was masked by the slow drift, even when partially accounted for by (log) onset time as a predictor.

To summarize this section, results for simulated time series were in qualitative and quantitative agreement with behavioral data showing a consistent increase of the shape parameter (more normal-like distribution) for higher levels of accumulated perceptual history (larger differences in self-adaptation levels). Our results for history-driven changes are in good agreement with prior work that fixed the dynamic regime for the entire experimental or simulation run, suggesting similar underlying changes in both cases.

### Regularity of dominance phases as a function of perceptual history

**Figure 8.**
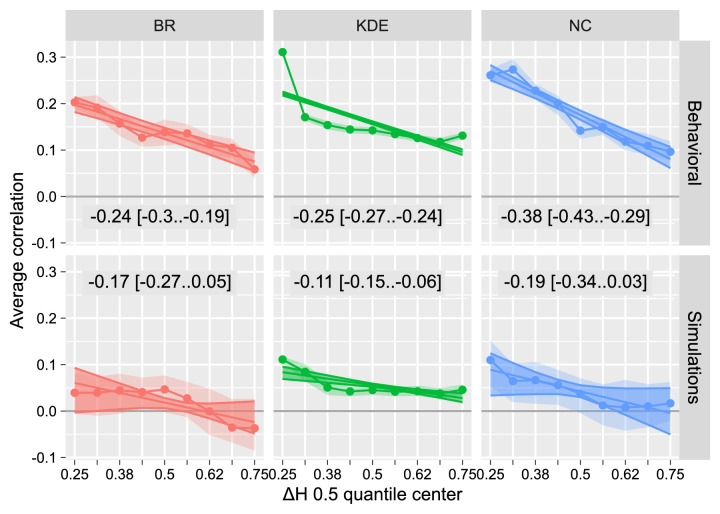
Correlation between predicted and observed dominance phase durations for dominance phases grouped based on the cumulative history difference. Data was split into nine overlapping quantiles of 50% width that moved at nine steps from centered at 0.25 (lower half) to 0.75 (upper half). Circles and faint stripes correspond to mean and 89% CI for posterior correlation between predicted and observed dominance phases. Straight lines and strong stripes, as well as in-plot text, show mean and 89% CI for fitted linear regression.

Our second question was whether dynamic changes in the balance between neural noise and self-adaptation consistently affect the regularity of dominance phase durations. To this end, we computed Pearson's correlation between observed and predicted dominance durations for different subsets of dominance phases based on the estimated accumulated perceptual history. Specifically, we used a sliding window that included 50% of dominance phases and moved it in nine steps from 0.25 (lower half) to 0.75 (upper half) and computed Pearson's correlation between observed and predicted dominance duration (similar to Figure 4B). Then, we fitted a linear regression separately for each display (BR, KD, and NC) and data source (behavioral and simulated) to estimate the dependence. For behavioral data, we observed a strong dependence with higher levels of accumulated perceptual history (larger differences in perceptual adaptation) associated with lower correlation (i.e. less predictable dominance phases; Figure 8, top row) and vice versa. The relationship was qualitatively similar but much weaker for the simulated data (bottom row in Figure 8).

Why would a larger difference in self-adaptation levels, the deterministic component of a typical computation model, lead to less regular dominance phases? The answer could lie in the duration of these phases. As established by prior work (Pastukhov & Braun, 2011) and confirmed by results in the section above, higher levels of accumulated perceptual history (larger difference in adaptation levels) lead to *longer* dominance phase durations. This, in turn, gives more opportunities for the noise to play its role and induces a perceptual switch. Conversely, comparable adaptation levels mean that the self-adaptation of a dominant state may quickly reach the switching threshold making them more regular. This idea is supported by the contrast data set (see Figure 6). Here, higher contrast is associated with stronger self-adaptation (hence, states reach the switching threshold faster), *shorter* dominance phases, and more regular dominance phases.

Note that it is the accumulated perceptual history (difference between adaptation levels) prior to the phase rather than the duration of the dominance phase itself that is predictive. In addition, note that the regularity of brief dominance phases could be even higher as they are more affected by variance in response times (Pastukhov, Vonau, & Braun, 2012) and, therefore, are noisier relative to the longer durations. It would be interesting to investigate such relative regularity using a better method for timing switches, such as an external clock (Pastukhov et al., 2012) or eye movements (Aleshin, Ziman, Kovács, & Braun., 2019).

At the same time, results that rely on spontaneous variability in percept durations might need to be interpreted differently than results that rely on experimentally varied variability. Our assumptions, consistent with those used in most models, is that self-adaptation speed and level of internal noise remain constant throughout the block. However, it is possible that both are modulated throughout the block by fatigue or (in)attention (Paffen & Alais, 2011; Pastukhov & Braun, 2007). In this case, duration of dominance phases may reflect the current speed of self-adaptation build-up and, independently, the regularity of dominance phases would depend on the current level of noise. In turn, this would mean that although a cumulative history measure, computed with the assumption of stable self-adaptation and noise, would be correlated with the duration of following dominance phase durations, it would not reflect the actual adaptation state and should not be interpreted causally. Therefore, further research that would combine both spontaneous and experimentally controlled variations in both self-adaptation and noise is needed to disentangle these effects.

To summarize this section, we found that higher levels of accumulated perceptual history (larger difference in self-adaptation states) led to less regular dominance phase durations, likely due to longer phase durations that allow for a stronger influence of noise. However, the exact relationship between accumulated perceptual history and regularity of dominance phase durations may depend on fluctuation of the adaptation process and the level of internal noise.

## Discussion

The aim of this study was to examine how minor changes in the balance between noise and self-adaptation due to the perceptual history influences the distribution of the following dominance phase durations. Specifically, we were interested in (1) changes to the overall distribution shape and (2) regularity of dominance phases. For the overall distribution shape, we observed a consistent change for the shape parameter of the Gamma distribution, meaning that higher levels of accumulated perceptual history (larger difference in self-adaptation levels) led to a more normal-like distribution. This was observed both for all stimuli and experimental conditions in behavioral data and for simulations generated by a spiking model of bistability (Laing & Chow, 2002). Concerning the effect of perceptual history on the regularity of dominance phases, we found that its higher levels (larger differences in self-adaptation levels) made dominance phases less regular.

The reported pattern was consistent for three multistable displays that are thought to rely on different underlying neural networks (Brascamp et al., 2018; Brascamp et al., 2019; Pastukhov et al., 2019). Thus, they are likely to generalize to other multistable stimuli including other modalities (Denham, Bendixen, Mill, Tóth, Wennekers, et al., 2012), and can serve as an additional check on whether a visual experience similar to multistability taps into similar neural mechanisms. For example, there exists a curious case of Kanizsa's anomalous transparency figure that produces bistable perception with times series that are overall similar to those of other bistable displays, yet, it differs substantially in its serial dependencies during continuous as well as intermittent presentations (Kogo, Hermans, Stuer, van Ee, & Wagemans, 2015). In cases like these, the two additional characteristics of multistability – consistent history-dependent changes in distribution shape and dominance durations regularity – could serve as additional ways to identify reasons for possible dissociations.

Our results on changes in the distribution shape are in good agreement with prior work that fixed the dynamic regime for the entire experimental (Brascamp et al., 2009; van Ee, 2009) or simulation (Pastukhov et al., 2013) run. This suggests similar underlying changes in the balance between self-adaptation and the noise in both cases and extends them to a dynamic case. The advantage of our analysis is that it allows characterizing this dependence and, therefore, changes in the balance between self-adaptation and the noise also for stimuli, such as a Necker cube or face-vase perceptual rivalry, where its explicit manipulation is problematic. At the same time, the perceptual history approach might help to differentiate from the case when changes in dominance time distribution reflect other sources of instability. For example, increased dot density and velocity speed up perceptual alternations in kinetic-depth effect displays (Brouwer & van Ee, 2006). Although this speed-up looks similar to that caused by increased contrast in binocular rivalry (van Ee, 2009), it is explained primarily by changes to the scale parameter of the Gamma distribution alone. This hints at different reasons for increased switches such as its decreasing costs (Pastukhov et al., 2012). In cases like these, information on history-dependent changes within and stimulus-dependent differences between individual conditions could help to establish the overlap between effects.

Highly consistent history-dependent changes also provide more specific constraints for models of multistable perception. In prior work, models were commonly checked for serial dependencies (Laing & Chow, 2002) and for their ability to accurately reproduce central moments of the distribution (Cao et al., 2016; Cao et al., 2021). Our results build on that as they allow us to characterize dynamic history-dependent changes and identify specific groups of dominance phases based on expected levels of self-adaptation. Our results are strongly linked to the idea of a dynamic interplay between noise and self-adaptation, as they can be accounted for even by a relatively simple model built on this idea (Laing & Chow, 2002). Comparable results would likely be obtained from the predictive coding framework that uses the same overlap principles but a different conceptualization as an interplay between stability prior and noisy evidence (Weilnhammer et al., 2017). However, it would be an interesting test for other conceptualizations, such as the Necker-Zeno Model for Bistable Perception (Kornmeier et al., 2017), models based on Bayesian sampling (Moreno-Bote, Knill, & Pouget, 2011), or attention-based models (Li, Rankin, Rinzel, Carrasco, & Heeger, 2017).

The fact that larger differences in levels of self-adaptation (as estimated through the accumulated perceptual history measure) lead to longer and more variable dominance phases has interesting practical implications for research on the causes of perceptual switches. Switching dynamics include other influences, most notably top-down attention (Dieter, Brascamp, Tadin, & Blake, 2016). However, its ability to exert influence depends on the multistable display (Meng & Tong, 2004) and its specific configuration (Brouwer & van Ee, 2006). The larger differences in the self-adaptation lead to longer recovery times before the switching threshold is reached. That in turn might provide a larger window of opportunity for attention to exert its influence and induce a perceptual switch. This idea gives interesting testable predictions for the timing and direction of the visual processing cascade that resolves perceptual ambiguity (de Jong, Vansteensel, van Ee, Leijten, Ramsey, et al., 2020), as well as the closer link between the processing of exogenous changes and self-adaptation rather than attention-driven endogenous switches. Future studies could also explore the effects of accumulated history on percept durations and predictability in more detail by using tasks that allow for a more precise control of adaptation levels and the perceptual noise (Kim, Grabowecky, & Suzuki, 2006). This could help to disentangle the effects of accumulated history from those of the internal noise and self-adaptation levels on the temporal dynamics of the perceptual alternations.

Finally, our results serve as a reminder that durations of individual dominance phases for multistable perception time series are not independent and identically distributed. Of course, this conclusion is not perfectly new (Pastukhov & Braun, 2011; van Ee, 2009), even if initial reports assumed such a memory-less process (Borsellino et al., 1972; Levelt, 1967). Nonetheless, an average dominance duration or, inversely, switch rate, often serves as a main measure of interest, particularly when comparing different groups of participants (e.g. Díaz-Santos, Mauro, Cao, Yazdanbakhsh, Neargarder et al., 2017; Kornmeier et al., 2017; Ukai, Ando, & Kuze, 2003). As illustrated in Figure 2, for a skewed distribution the same average dominance duration may correspond to a different underlying distribution shape with a different balance between self-adaptation and the noise (Arani, van Ee, & van Wezel, 2018; van Loon, Knapen, Scholte, St John-Saaltink, Donner et al., 2013). Our results suggest that even if groups are different in their alternation rate, it is always a promising idea to take a closer look at the distribution itself (e.g. by looking at its central moments; Cao et al., 2021), as well as serial dependencies (Hudak, Gervan, Friedrich, Pastukhov, Braun et al., 2011). The freely available R package *bistablehistory* (Pastukhov, 2022) used in the current study provides a convenient automatic tool for such in-depth fitting. It offers information on the time constants of the self-adaptation process for individual participants and its effect on individual dominance phase durations. Such an analysis is particularly beneficial when comparing observers’ groups (Hudak et al., 2011). This is the reason for us to encourage the interested reader to give it a try!

## Conclusions

We presented a novel analysis method that fits a Gamma distribution considering a simultaneously fitted estimate of cumulative perception history. We used it to demonstrate two previously unreported features that were present in all analyzed multistability time series both for behavioral and simulated data. Both of these features are linked to an increased prior cumulative history (larger difference in self-adaptation levels) as they shifted the shape of the distribution toward a more normal-like distribution and reduced the regularity of individual dominance phases, making them less predictable. Our results together with a freely available implementation of the analysis method pave way for a finer analysis of individual dominance phases within time series. They also remind us that dominance phase durations in multistable perception are not independent and identically distributed.
